# Validation of echocardiographic indices of right ventriclular systolic function with cardiac magnetic resonance: a comparative study

**DOI:** 10.1186/1532-429X-13-S1-O75

**Published:** 2011-02-02

**Authors:** Suchi K Grover, Darryl P Leong, Payman Molaee, Mitra Shirazi, Adhiraj Chakrabarty, Amy Penhall, Rebecca Perry, Majo Joseph, Joseph B Selvanayagam

**Affiliations:** 1 Flinders Medical Centre, Bedford Park, Australia; 2 Royal Adelaide Hospital, North Terrace, Australia

## Introduction

Right ventricular ejection fraction (RVEF) is an important predictor of outcome in heart failure patients. Although cardiac magnetic resonance (CMR) assessment of RV function is considered gold standard and echocardiographic measurement of RVEF is challenging due to its unique geometry; CMR still has limited availability in the wider community. Therefore alternative echocardiographic indices such as tricuspid annular plane systolic excursion (TAPSE), peak tricuspid annular systolic velocity (RV S’) and RV fractional area change (RV FAC) have been evaluated and demonstrated prognostic value, however, comparison studies with RVEF by CMR are limited.

## Purpose

Our objective was to evaluate echocardiographic indices of RV function such as TAPSE, RV S’, and RV FAC and compare these with CMR assessment of RVEF and patient functional status.

## Methods

We prospectively recruited 73 subjects: 56 with newly diagnosed systolic heart failure (age 55±27 years) and 17 healthy controls (age 53±8 years). Subjects underwent transthoracic echocardiography, CMR, both under similar loading conditions; and 6-minute walk test. Echocardiographic E and A velocities, TAPSE, RV S' and RV areas were measured in the conventional manner. Ventricular volumes were measured from cine CMR images by planimetry.

## Results

TAPSE exhibited the strongest correlation with CMR RVEF (r^2^ = 0.42, p <0.001). CMR RVEF could be estimated by the equation: CMR RVEF = (17.7 x TAPSE ) + 17, where TAPSE is measured in centimetres. RV S’ and RV FAC demonstrated only moderate correlation with CMR RVEF (r^2^ = 0.23, p<0.001 and r^2^ = 0.28, p = 0.002 respectively). ROC curve analysis for optimal cut-off values of RV indices in the estimation of CMR RVEF <45% are displayed in table 1.

**Table 1:** ROC curve analysis of RV indices

Parameter	Cut-off	Sensitivity	Specificity	AUC (95% CI)
TAPSE	<1.7cm	92%	62%	0.78 (0.62 - 0.93)
RV S'	<7.4cm/s	84%	50%	0.77 (0.65 - 0.88)
RV FAC	<48%	81%	50%	0.56 (0.32 - 0.8)

Left ventricular ejection fraction by CMR (r^2^ = 0.18, p = 0.001), CMR RV end-diastolic volume (r^2^ = 0.08, p = 0.03), RV S’ (r^2^ = 0.15, p = 0.004) and E’ (r^2^ = 0.15, p = 0.001) were significantly associated with 6-minute walk distance, whereas CMR RVEF, TAPSE, RV FAC, transmitral E wave velocity, E/A ratio and E/E’ were not. By multiple regression analysis, left ventricular ejection fraction was the only independent predictor of 6-minute walk distance (p = 0.001).

## Conclusions

TAPSE by echocardiography most strongly correlated with RVEF by CMR; RV S’ and RV FAC exhibited only moderate correlation. RV S’ was the only index of RV function that was associated with functional capacity by 6-minute walk test.

